# The role of radiotherapy in newly diagnosed primary CNS lymphoma: A descriptive review and a pragmatic approach to clinical practice

**DOI:** 10.1016/j.ctro.2022.12.002

**Published:** 2022-12-09

**Authors:** Venkada Manickam Gurusamy, Saju Raveendran Divakar, Suparna Halsnad Chandramouli, Beena Kunheri, Hissa Hussain Al-Abdulla, Ghazia Shaikh, Rajiv Chaudary Apsani, Mohamed Riyaz Poolakundan, Palmira Caparrotti, Rabih Wafiq Hammoud, Noora Al-Hammadi

**Affiliations:** Department of Radiation Oncology, National Center for Cancer Care and Research (NCCCR), Hamad Medical Corporation, Doha, Qatar

**Keywords:** Radiotherapy, PCNSL, Chemotherapy, Stem cell transplantation, Low-dose radiotherapy

## Abstract

Earlier, prior to the development of effective systemic therapy, monotherapy with whole-brain radiotherapy (WBRT) was widely used to treat primary central nervous system lymphoma (PCNSL). Recently, chemotherapy, especially with high dose methotrexate (HDMTX), has largely replaced WBRT as upfront treatment, and the most accepted standard of care is induction with a combination drug therapy followed by consolidation therapy with either autologous stem-cell transplantation (ASCT) or radiation. Whilst WBRT is an effective component of treatment, it is occasionally associated with risk of permanent, irreversible neurotoxicity when doses of more than 30 Gy are used. Hence, there has been a strong focus on the optimization of radiotherapy (RT) which includes dose reduction in the consolidation phase. In this comprehensive review, we have summarized the progress on clinical results and evidence considering the role and use of radiation including combined treatment modalities, low-dose radiotherapy, and neurotoxicity. Finally, we present a practical approach to low-dose WBRT and boosting higher doses to the gross tumor that can be integrated into clinical practice.

## Highlights

• The most accepted standard of care for primary central nervous system lymphoma (PCNSL) is induction with chemotherapy followed by consolidation therapy with either autologous stem-cell transplantation (ASCT) or radiation.
• Monotherapy with whole-brain radiotherapy (WBRT) is often used to treat patients who are ineligible for chemotherapy.
• WBRT is occasionally associated with risk of permanent, irreversible neurotoxicity when doses of more than 30 Gy are used.
• There has been a strong focus on low dose radiotherapy in the consolidation phase.

## Introduction

The 2017 World Health Organization classification of hematopoietic and lymphoid tumors recognized primary diffuse large B-cell lymphoma of the central nervous system (PCNSL) as a distinct entity [1]. PCNSL is a rare and aggressive lymphoproliferative disease accounting for <1 % of all non-Hodgkin lymphomas and 4 % of all brain malignancies but its incidence appears to be increasing, especially in the elderly population [2], [3].

Earlier, prior to the development of effective systemic therapy, monotherapy with whole-brain radiotherapy (WBRT) was widely used to treat PCNSL. Recently, chemotherapy, particularly with high dose methotrexate (HDMTX), has largely replaced cranial irradiation as upfront treatment, and the most accepted standard of care is induction with an MTX-based multi-agent regimen followed by consolidation therapy with either autologous stem-cell transplantation (ASCT) or radiation [4], [5]. WBRT alone is now reserved for salvage therapy for those who are ineligible for multiagent therapy with poor performance status [6]. The prognosis for PCNSL has significantly improved with this combined approach however overall treatment outcomes remain poor. Only a half of patients attain long-term survival, with age and performance status being the significant prognostic variables [7].

Although WBRT is considered an important component of the successful CNS lymphoma management, it is associated with risk of long-term toxicity, including severe progressive cognitive dysfunction at times [8]. Hence, there has been a strong focus on the optimization of radiotherapy (RT) which includes dose reduction in the consolidation phase. Particularly, over the past few years, prospective clinical studies introducing this new strategy have rapidly expanded the volume of data and literature and transformed the treatment landscape for patients who are ineligible or inaccessible for ASCT.

The main objective of this review was to provide a good overview of the scientific literature pertaining to the role and use of radiation in the treatment of patients with newly diagnosed PCNSL and to give recent updates on reduced dose radiotherapy. Here, we have summarized the progress of clinical evidence considering the combined modality treatment, low-dose radiotherapy, and neurotoxicity. Larger, multi-center phase II/III clinical trials are discussed in detail focusing on outcomes and toxicities while the role of radiation in a relapsed or recurrent setting is not part of this article. Finally, we present a practical approach to routine clinical practice pertaining to RT**.**

## Methods

An extensive search for articles published from January 1990 to January 2022, and available in PubMed, and Google Scholar was performed. Search terms included “PCNSL”, “methotrexate”, “radiotherapy”, “reduced dose radiotherapy” “whole brain radiotherapy” and “neurocognition.” Original peer-reviewed articles published in English and reporting the results of outcomes in adult patients were eligible for inclusion. Abstracts and full texts were screened manually and were included in this review if radiotherapy was part of treatment. Case studies and articles including <10 patients were excluded.

## Whole-brain radiotherapy

Only a few studies have critically evaluated the prominent role of WBRT as a primary modality of intervention. Importantly, in a prospective study of patients with PCNSL who were 70 years and older, WBRT along with methylprednisolone was employed until progression. Patients received 41.4 Gy to the whole brain and an additional 9 Gy boost to gross disease. Nineteen patients were included in the study and 3 patients had complete response (CR) with therapy and 5 patients experienced a partial response (PR) or regression. The 6-month overall survival (OS) was 37 %, and the median survival was 5.5 months [9]. In addition, the Radiation Therapy Oncology Group (RTOG) conducted a prospective phase II study in which forty-one patients were irradiated with the dose of 40 Gy and a 20 Gy boost to the tumor plus 2 cm margin. The median overall survival was 11.6 months, with 48 % of the patients surviving at 1 year and 28 % surviving at 2 years. Six patients (15 %) survived without evidence of disease at the last follow-up visit with a median survival of 53.9 months (range 8.8–67.2 months). Twenty-one (51 %) failed in the brain alone and brain failures occurred solely within the original tumor volume within the treatment field with four exceptions. Karnofsky Performance Status and age were significant prognostic factors, being associated with poor survival [10].

Another study compared WBRT with best supportive care in patients with poor performance status and/or old age. A total of 48 patients were selected for analysis, among which 31 (64.6 %) patients received WBRT and 17 (35.4 %) received supportive care only. Median age was 74 years (range 30–89 years), and median follow-up among the survivors was 14.9 months (range 2.6–62.3 months). The median dose to the whole cranium was 30 Gy (range 10–52 Gy) which was associated with an improved median OS compared with supportive care (8.0 months vs 3.3 months, p = 0.005). In the subgroup analysis, a higher radiation dose to the whole brain was not associated with survival, but the addition of a boost to the gross tumor was correlated with improved survival [11].

## Combination of chemotherapy and WBRT

Observing the dismal outcomes after radiotherapy, a combination of chemotherapy and RT was designed and rigorously tested, aiming to enhance the effectiveness of therapy and survival. Particularly, a multicenter RTOG phase II trial prospectively studied the combined approach in which ninety-eight assessable patients received a HD-MTX-based regimen, following this WBRT was administered to the total dose of 45 Gy with cytarabine. The median progression-free survival (PFS) was 24.0 months, with 58 % of the patients having a complete response (CR) prior to RT. The median survival was 50.4 months in patients younger than 60 while it was 21.8 months in those aged 60 or older (P < 0.001). The overall survival was 32 % and the PFS was 25 % at 5 years. Twelve patients (15 %) experienced severe delayed neurologic toxicities characterized primarily as leukoencephalopathy. Eight (10 %) of these toxicities were fatal. There was no significant difference in Mini-Mental State Examination (MMSE) scores at 8 months after therapy in comparison to that at baseline [12].

A European Organization for Research and Treatment of Cancer Lymphoma Group Phase II Trial utilized HDMTX-based chemotherapy followed by radiation in a multicenter setting. Fifty-two patients were included, and 42 patients received radiation with a whole-brain dose of 40 Gy. The overall response rate was 77 % when the median estimated overall survival was 46 months. Kaplan-Meier 2- and 3-year survival estimates were 69 % and 58 %, respectively. The 10 % toxic death rate was observed during treatment and one death was related to late leukoencephalopathy [13]. Also, one of the largest phase III randomized studies evaluated the role of WBRT after completion of HDMTX. It hypothesized that the omission of radiation would not compromise overall survival (OS), using a noninferiority design where the dose was 45 Gy in 1.5 Gy per fraction. From 1999 to 2009, 551 patients (median age 63 years) were included. With a median follow up of 81.2 months, there was a prolongation of PFS by radiation (15.4 vs 9.9 months, p = 0.034) in the intent-to-treat population (n = 410) whereas no difference in OS was found (32.4 vs 36.1 months, hazard ratio [HR] 0.98, 95 % CI 0.79–1.26). In the as-treated analysis, WBRT significantly improved PFS in patients with complete response (HR 0.64, 95 % CI 0.44–0.94), but not OS (HR 0.93, 95 % CI 0.68–1.53). In patients without complete response, there was a significant difference in PFS among patients treated with WBRT (15.9 months) and no further therapy (8.9 months); HR 0.47 (0.95 % CI 0.35–0.62) [14]. However, this study failed to prove its primary hypothesis, showing poor protocol adherence, which is the main criticism of this study. Approximately 10 % of patients were lost to follow-up. Crossover was another important violation of the study protocol. Only 65 % of patients who achieved CR in the WBRT group received intended therapy, which can significantly impact the conclusions from this study.

Then, the focus and attention of research was gradually shifted to using autologous stem cell transplantation (ASCT) as consolidation therapy. Importantly, comparison of WBRT with ASCT was done in patients aged 70 years or younger in the International Extra nodal Lymphoma Study Group-32 (IELSG32) study, an international phase II randomized trial. Of the 122 patients eligible for randomization, 118 patients were assigned to receive WBRT or ASCT. The dose was 36 Gy in 20 fractions, with the addition of a 9 Gy tumor-bed boost in patients who had a partial response (PR); orbits were shielded after 30 Gy. The 7-year PFS was 55 % (95 % CI = 50–60 %) for WBRT and 50 % (95 % CI = 43–56 %) for ASCT (HR: 1.15, 95 % CI: 0.78–1.68; p = 0.46), and the 7-year OS was 63 % (95 % CI = 60–65 %) and 57 % (95 % CI = 53–61 %), respectively (HR: 1.25, 95 % CI: 0.84–1.88; p = 0.26). Both therapies were well tolerated however two toxic deaths were recorded in the ASCT arm. At a median follow-up of 88 months, 51 patients’ disease experienced relapse. At progression or relapse, lymphoma involved the primary site of disease in 97 % of cases (113/117). Both WBRT and ASCT were active and the comparable efficacy of both therapies was confirmed. A remarkable increase in the CR rate was achieved after induction therapy, with a CR of 95 % after irradiation and 93 % after transplantation [15].

Additionally, patients who were 18–60 years of age were randomly assigned to receive WBRT or ASCT after induction with an MTX-based regimen in the PRECIS study. While 140 patients were recruited from 23 French centers, the radiation dose consisted of 40 Gy over four weeks. The median follow-up was 98 months. In per-protocol analysis, event-free survival (EFS) at 8 years was 67 % (95 % CI = 55 %–83 %) in the ASCT group vs 39 % (95 % CI = 27 %–57 %) in the WBRT group (P = 0.03). Relapse occurred in 3 patients in the ASCT group vs 24 in the WBRT group (HR = 0.13, P < 0.001). Overall survival at 8 years was 69 % (95 % CI = 57 %–85 %) in the ASCT group vs 65 % (95 % CI = 52 %–81 %) in the WBRT group (P = 0.90). A total of five patients died from ASCT-related toxicity, and four died from WBRT-related toxicity. The stable/progressive disease at the end of induction treatment was significantly correlated with shorter EFS and OS [16]. The study concluded that 40 Gy WBRT should be avoided in first-line treatment because of its neurotoxicity and suboptimal efficacy in reducing relapses while ASCT appeared to be highly efficient in preventing relapses. Unexpectedly, a greater percentage of PRECIS patients had residual disease after consolidation in comparison to the IELSG32 study (42 % vs 6 %). Overall, we could see that addition of ASCT or WBRT with induction chemotherapy has resulted in dramatic improvement in treatment outcomes and 5-year overall survival thus in recent time, this approach is primarily employed, especially in younger and fit patients.

## Reduced dose radiotherapy

Some single-center studies proposed low-dose radiotherapy would be as effective as high-dose WBRT after completion of systemic therapy in terms of controlling the disease [17]. Moreover, lowering the dose can better reduce severe late effects and toxicities. The impact of this novel and useful approach has further been evaluated in large randomized trials in recent years. Notably, a multicenter phase II study was conducted to assess the efficacy of reduced dose WBRT at the end of chemotherapy. Those achieving a CR received 23.4 Gy, otherwise, a standard dose (45 Gy) was offered. Fifty-two patients were enrolled, and thirty-one patients (60 %) achieved a CR and received the low dose. With a median follow-up of 5.9 years, the 2-year PFS was 77 %. Strikingly, the median PFS was 7.7 years and the median PFS was not reached in patients younger than 60 years and was 4.4 years in patients ≥ 60 years. The 5-year OS was 80 % (95 % CI, 66 % to 94 %) [18]. Further, a multicenter phase II study examined the age-adapted chemotherapy protocol followed by radiotherapy. A total of 99 patients were recruited and all of them received RT with 20 Gy whole-brain RT and a 30 Gy boost to the tumor bed, in 2-Gy fractions. At completion of the entire chemotherapy plus RT treatment, CR was observed in 49 (49 %) patients, PR in 19 (19 %), and PD in 8 (8 %). With a median follow-up of 83 months, the median OS was 33 months and the 5-year OS rate was 34 % [19].

An intergroup phase III randomized study investigated the efficacy of adding immunotherapy to chemotherapy and recruited 200 patients (109 men and 91 women). Patients aged 60 years or younger with a response at the end of chemotherapy received high-dose cytarabine and low-dose WBRT. Patients with a CR received 30 Gy in 20 fractions; an integrated boost of 0·5 Gy was simultaneously administered to the tumor area in patients with a PR. The median survival was 56·7 months, with 3-year OS being 58 %. The progression-free survival was 58 % (95 % CI 48–67) at 1 year and the median overall survival in patients aged 60 years or younger was 56.7 months (95 % CI 56.7 - not reached) [20]. Moreover, an RTOG phase II study randomized patients to receive MTX with low-dose WBRT (chemoRT arm) with a dose of 23.4 Gy versus chemotherapy alone. Among eligible patients, 43 were enrolled in the chemoRT arm and 44 in the chemo arm. At a median follow-up of 55 months, the 2-year PFS was 54 % (chemo) and 78 % (chemoRT), with data still maturing [21].

Recently, a phase III study of HDMTX and WBRT with (arm B) or without (arm A) temozolomide was conducted in Japan. After the induction phase, 122 were randomly assigned with arms A and B. The radiation dose was 30 Gy ± 10 Gy boost, with the 2-year PFS being 60.6 % in arm A and 49.9 % in arm B [22]. Even though above-mentioned trials were not designed to evaluate the efficacy of low-dose radiotherapy or the quality of life (QoL) after the treatment as compared to higher doses, they have reduced WBRT doses in the consolidation phase to avoid or minimize neurotoxicity, and long-term follow-up has shown that treatment outcomes are comparable to historical controls. This approach can be considered a treatment option in the consolidation phase after induction chemotherapy, especially in a resource constraint setting where ASCT is not a choice.

## Neurotoxicity

The primary tumor itself has a great detrimental effect on the cognitive function and quality of life of patients with PCNSL [23], [24]. Cognitive impairment, walking disorders, neurological deficits, and poor performance status are the common clinical features at the time of presentation [23]. Treatment-related neurotoxicity is a common but serious complication among long-term survivors and has been a significant concern for decades because whole-brain RT is often employed instead of local or tumor focused radiation. Few patients experience severe cognitive difficulties and dementia that can interfere with their daily functional ability in comparison to pre-diagnosis levels [24], [25], [26]. When an intense combination of radiotherapy and HDMTX is used, toxicity rates can potentially increase in comparison to chemotherapy alone [24]. Omuro AM et al, reported that although leukoencephalopathy is a delayed reaction, some of their patients had developed symptoms within months of completing treatment in a single-center study. One hundred eighty-three patients were included in the analysis and neurotoxicity developed in 43 patients. However, medical records from 30 of the 43 patients with neurotoxicity contained sufficient information to evaluate the clinical course of neurotoxicity. The 5-year cumulative incidence of neurotoxicity was 24 %, and 67 % of the patients who developed neurotoxicity had moderate or severe gait disturbance at 2-year follow-up. The presentation included rapidly progressive subcortical dementia characterized by psychomotor slowing, memory dysfunction, behavioral changes, gait ataxia, and incontinence. Univariate analysis showed that age of 60 years or older, female sex, presence of mental status abnormalities at diagnosis, and cranial radiotherapy were the potential risk factors with significant p-values. Only radiotherapy was a significant risk factor in the multivariate analysis [26].

However, tolerable delayed side effects without seriously affecting the daily activities of most patients treated with radiation have been reported in prospective studies. Cognitive impairment was found in 12 evaluable patients (63 %) who received WBRT despite a complete tumor response in an EORTC study. Four patients (21 %) showed severe cognitive deficits, and the percentage of impaired test indices correlated with age. In comparison, only two control subjects (11 %) who received chemotherapy alone showed cognitive dysfunction [27]. Furthermore, in the GPCNSLSG-1 study, 318 out of 551 patients were included in the per-protocol population. Quality of life scores and MMSE scores were measured at baseline and yearly up to 4 years in that population. Cognitive functioning and global health status were reduced in the early WBRT arm as compared to the no early WBRT arm at 2 years of follow-up. Also, the MMSE testing revealed lower score values in the early RT arm, with fatigue, appetite loss and hair loss being more intense [28].

ASCT as a consolidation therapy has resulted in fewer neurocognitive toxicities in comparison to WBRT. Particularly, neuropsychological test scores recorded immediately after treatment and at the last visit of follow-up were analyzed in the IELSG 32 study. Significant impairment of some attentive/executive functions among patients treated with WBRT was observed, contrasting with significant improvement in these attentive/executive functions, memory and EORTC quality-of-life figures in patients treated with ASCT. MMSE did not show a significant difference between consolidation arms. However, only 45 % of the randomized patients were included for cognitive functions and quality of life analysis [15]. Moreover, in the PRECIS study, neurocognitive evaluations were performed in both arms throughout the follow-up at a predefined interval until a relapse occurred. Mean scores of Mattis Dementia Rating Scale and MMSE declined after WBRT in the global cognitive assessment whereas they were stable after ASCT. Scores of episodic verbal memory, executive function and psychoaffective status (motivation) were also showed the similar trend. Compared with the ASCT group, more patients in the WBRT group had significant deteriorations in balance (52 % vs 10 %, P = 0.001) and neurocognition (64 % vs 13 %, P < 0.001) during follow-up. Ischemic strokes were reported in seven patients after WBRT, including two patients with no vascular risk factors. The percentage of patients included for neurocognitive testing decreased overtime in both arms [16].

It seems that reduced dose RT can lower the development of neurocognitive toxicity and other neurological sequelae to a significant extent during follow-up. Specifically, twelve of the 32 patients who had received 23.4 Gy completed neuropsychological evaluations up to 48 months in a phase II trial and there was no evidence of cognitive decline however decreased motor speed was noticed (p < 0.05). Minor fluctuations were observed in memory performance over time whereas depressed mood was not reported, and self-reported quality of life remained stable [18].

Moreover, forty-three irradiated patients were assessed for neurocognitive functioning up to 2 years postradiotherapy in the HOVON 105/ALLG NHL 24 trial. The results showed no noticeable and clinically relevant changes over time, neither improvement nor deterioration, in neurological function including attention/ executive functioning, information processing, motor speed and memory. However, a decrease in information processing speed was seen in 11 % of patients at 24 months follow-up. There were inverse associations between white matter abnormalities and brain atrophy on magnetic resonance imaging (MRI) and neurocognitive functioning scores. Although those correlations were statistically significant in all domains except in memory for white matter changes, the changes in neurocognitive scores were rather small, resulted in only a small, not clinically relevant, deterioration in neurocognitive functioning. For brain atrophy, a 10 % decrease of brain volume was significantly associated with a deterioration in memory (p = 0.027). Other associations were not significant or clinically relevant. Overall, in the first 2 years posttreatment, a lower dose of RT was not harmful to neurocognitive functioning, compared to immediately after WBRT [29].

Results of selected studies that utilized RT as part of treatment are summarized in Table 1. Clinical outcomes including overall survival and neurotoxicity are broadly outlined for comparison. Neurotoxicity analysis associated with radiation continues to be defined. The cognitive assessment methods or scores utilized in above mentioned prospective trials or retrospective studies were highly heterogenous, which makes difficulties in deriving a conclusion. The importance of other confounding factors that likely alter cognitive status, such as age, atherosclerosis, radiation dose, dose per fraction and usage of antiepileptic drugs or chemotherapy regimens were also not evaluated in detail. Moreover, a significant proportion of the study population was not included for neurocognitive analysis in almost all studies due to various reasons including progressive disease or relapse. Cognitive evaluation performed by trained neuropsychological teams is also essential in this toxicity analysis. These factors must importantly be considered while looking treatment-related toxicities affecting QoL and making treatment decisions in this patient population.

**Table 1:** A summary of selected studies that included radiotherapy in the therapeutic management of newly diagnosed PCNSL.

Study	Methodology	HD-MTX	Results	Neurotoxicity
Laack NN 2006 [9]	Prospective study N = 19 Age: 70–83 yrs; 41.4 Gy + 9 Gy boost in 1.8 Gy per fraction	No chemo	6-month OS: 37 %; Median OS: 5.5 mo	Decrease in MMSE scores n = 2 Decrease in performance score n = 10
Nelson DF 1992 [10]	Prospective trial N = 41 Age: <60 yrs n = 14 ≥ 60 yrs n = 27; 40 Gy + 20 Gy boost in 1.8 Gy per fraction	No chemo	2-year OS: 28 %; Median OS: 11.6 mo	Severe neurotoxicity encephalomalacia, brain necrosis n = 2
Ferreri AJM 2022 [15]	IELSG 32 phase II trial WBRT N = 55 Age 58 (18–70) yrs; 36 Gy in 1.8 Gy per fraction + boost 9 Gy vs ASCT N = 58 Age 58 (26–70) yrs	Yes	7-year PFS 59 % vs 50 %; 7-year OS 63 % vs 57 %	Significant impairment in attentive/executive functions with WBRT
Houillier C 2022 [16]	PRECIS phase II trial WBRT N = 53 Age 54.5 (22–60) yrs; 40 Gy in 2 Gy per fraction vs ASCT N = 44 Age 55 (25–60) yrs	Yes	8-year EFS: 67 % vs 39 %; 8-year OS: 69 % vs 65 %	Balance decline 52 % and severe troubles 33 % Cognitive decline 64 % and severe troubles 39 % Stroke n = 7 after WBRT
Ghesquieres H 2010 [19]	Phase II trial N = 99 Age: 63 (20–83) yrs; 20 Gy + 30 Gy boost in 2 Gy per fraction.	Yes	5-year OS: 34 %; Median OS: 33 mo	Grade 2–3 leukoencephalopathy – 32 %. Death n = 5
Bromberg JEC 2019 [20]	HOVON 105/ALLG NHL 24 phase III trial N = 200 Age 61 (56–66) yrs; 30 Gy in 1.5 Gy per fraction ± SIB boost 10 Gy	Yes	1-year PFS: 58 %; 3-year OS: 58 %	No clinically relevant changes over time after WBRT in attention/ executive functioning, information processing, motor speed and memory [29]
Kwak YK 2017 [30]	Retrospective study N = 32 Age: < 60 yrs n = 12; ≥ 60 yrs n = 20; 30 Gy (range, 14.4–50) in 1.8–2 Gy per fraction.	No chemo	Median PFS: 15.8 mo; Median OS: 16.3 mo. Symptomatic improvement 84.4 %	White matter changes − 21.8 % No grade 4 toxicity
Iwabuchi M 2016 [31]	Retrospective study N = 24 Age: 66 (22–90) yrs; Partial brain RT 54 Gy in 1.8–2 Gy per fraction	With or without MTX	3-year PFS 68 %; 3-year OS 60 %	Neurocognitive dysfunction n = 7
Shah GD 2007 [32]	Phase II trial N = 30 Age: 57 (30–76) yrs; 23.4 Gy if CR; others 45 Gy in 1.8 Gy per fraction	HD-MTX	2-year PFS 67 %; 2-year OS 57 %	No neurotoxicity observed in comparison with baseline.
Pottgen C 2003 [33]	Retrospective study N = 14 Age: 22–76 yrs; 39.6–50.4 Gy + boost 10.6 Gy in 1.8 Gy per fraction	Yes	5-year OS 42 %	Dementia n = 1 Visual loss and urinary incontinence n = 1 Memory loss n = 2
Chalice L 2017 [34]	Retrospective study N = 35 Age: 61 (19–71) yrs; 40 Gy + boost 20 Gy in 2 Gy per fraction	With or without HD-MTX	Median OS: 47.8 mo; 2-year PFS 62.3 %	Grade 3 luekoencephalopathy − 32 %. Mild to moderate cognitive deficit n = 6
Adhikari N 2018 [35]	Phase II trial N = 19 Age: 52.5 (31–67) yrs; 23.4 Gy if CR. Others 45 Gy in 1.8 Gy per fraction	Yes	Median OS: 19 mo; 2-year PFS 50.2 % 2-year OS 48.5 %	Improvement in neuropsychological assessments at 6 months after therapy.
Lee TH 2020 [36]	Retrospective study N = 20 Age: 58.7 (46–75) yrs; 21.6 Gy + boost up to 45 Gy in 1.8 Gy per fraction N = 60 Age: 56.1 (23–75) yrs; 30.6 Gy + boost up to 50.4 Gy	Yes Yes	3-year OS 77.8 %; 3-year PFS 53.3 %. 3-year OS 58.3 % 3-year PFS 45.8 %	Neurotoxicity ≥ Grade 2–6.9 % Neurotoxicity ≥ Grade 2–37.3 %
Kim N 2021 [37]	Retrospective study N = 40 Age: 55 (47–63) yrs; 23.4 Gy + SIB up to 40 Gy in 1.8 Gy per fraction vs no-RT N = 40	Yes Yes	2-year PFS: 56.6 % vs 53.8 % 2-year OS: 81.7 % vs 75.3 %	3-year neurotoxicity − 21.2 % vs 20.2
Koh HK 2017 [38]	Multicenter retrospective N = 26 Age: greater than 20 yrs; 30.6 Gy (18–45). Boost up to 50.4 Gy (30–59.4) in 1.2–3 Gy per fraction Chemo RT n = 179	No chemo Yes	RT alone Median OS: 15 mo; 5-year OS 22.2 %. Chemo RT Median OS: 77 mo; 5-year OS 56.2 % 5-year PFS 43.2 %	For both groups: neurotoxicity − 34.4 %. Grade 3 toxicity including leukoencephalopathy, memory impairment, altered mentality, dysphasia, and brain necrosis − 6.4 %.
Chanswangphuwana C 2018 [39]	Retrospective study N = 12 Age: 56 (16–78) yrs; 30–36 Gy + Boost up to 45–50 Gy in 1.8 Gy per fraction	Yes	3-year PFS 78.7 %; 3-year OS 87.5	Dementia n = 1 Mild cognitive impairment n = 5. All lived independently and could perform their daily-life activities
Park JS 2017 [40]	Retrospective study N = 54 Age: 47(19–59) yrs; 36 Gy and 45 Gy + boost 9 Gy in 1.8 Gy per fraction	Yes	5-year OS 63.3 %; 5-year PFS 42.6 %. No difference in outcomes between 36 Gy and 45 Gy	Memory disability n = 10. All patients received 45 Gy.
Nakasu Y 2016 [41]	Retrospective study Chemo RT N = 23 Age: 66 (42–79) yrs; 40–50 Gy vs Chemo N = 26	Yes	Median OS: 80.6 mo vs 38.7 mo	Not reported
Kobayashi H 2019 [42]	Retrospective study N = 54 Age: 60.5 (31–78) yrs; 30 Gy + boost in 2 Gy per fraction	Yes	Median OS: 58.4 mo	Not reported
Wang Y 2013 [43]	Phase II trial N = 14 Age: 55 (33–68) yrs; 40 Gy in 2 Gy per fraction	Yes	Median PFS: 21.4 mo; CR 85.7 %	No toxicity observed
Hohaus S 2009 [44]	Retrospective study N = 41 Age: 59 (31–78) yrs; 40 Gy + boost 20 Gy in 2 Gy per fraction	Yes	5-year PFS 46 %; 5-year OS 24 %	Not reported
Piriyakhuntorn P 2021 [45]	Retrospective study N = 41 Age; 61 (55–68) yrs; 30–36 Gy + boost 10 Gy	Yes	Median OS: 46.5 mo; 2-year PFS 55.3 %; 2-year OS 62.6 %	Not reported
Glass J 2016 [46]	Phase I/II trial N = 53 Age: 57 (24–73) yrs; 36 Gy in 1.2 Gy BID	Yes	2-year PFS 63.5 %; 2-year OS 80.8	Significant cognitive decline n = 2 Median QOL scores improved at 3 years
Ferreri AJ 2009 [47]	Phase II trial N = 79 Age: 58 (27–72) yrs; 36–40 Gy + boost 9 Gy in 1.8 Gy per fraction.	Yes	3-year PFS 38 %; 3-year OS 46 %	MMSE scores improvement n = 10 impairment n = 3 stable n = 3
Alvarez-Pinzon AM 2021 [48]	Retrospective study N = 55 Age: 56.9 ± 13.3 yrs; 10–20 Gy in single fraction	Yes	Median OS: 52.5 mo; Local control: 100 %	No clinical or radiological toxicity. There was a direct relationship between 2-year survival and standard dose increase.
Wu J 2018 [49]	Phase II trial N = 52 Age: 56 (20–69) yrs; 30 Gy + boost 15 Gy in 2 Gy per fraction.	With or without HD-MTX	2-year PFS 39 %; 3-year OS 51 %	Delayed neurotoxicity n = 9.
Ferrari AJ 2002 [50]	Retrospective study Primary RT N = 98 Age: 21–88 yrs; 45 Gy + boost Chemo RT N = 197 Age: 14–77 yrs; 36 Gy + boost	Yes	2-year OS 25 %; 2-year OS 45 %	Overall neurotoxicity − 21 % including loss of memory, weakness, extrapyramidal symptoms, depression. RT alone − 18 %, chemoRT − 28 %. Leukoenphalopathy, aphasia n = 7. Death n = 3
Besssell EM 2002 [51]	Two phase II trials N = 57 Age: 59 (21–70) yrs; 30.6–45 Gy in 1.8 Gy per fraction	With or without HD-MTX	5-year OS 55 %; 3-year risk of relapse: 44 %	No neurotoxicity observed with 30.6 Gy. Dementia n = 6 (60–69 years old). Mild cognitive dysfunction n = 1
Sheu T 2018 [52]	Retrospective study N = 22 Age: 60 (31–77) yrs 23.4 Gy (range, 23.4–36) ± boost up to 37.8 Gy (30–45) in 1.8 Gy per fraction	Yes	Median OS: 52.3 mo; Median PFS: 52.3 mo	No significant difference in neurocognitive scores. Brain necrosis n = 1
Abrey LE 2000 [53]	Retrospective study N = 52 Age: 65 (27–89) yrs. Twenty older patients deferred RT 45 Gy	Yes	5-year PFS 50 %	Late neurotoxicity − 25 %. Most of them older than 60.
Obrien P 2000 [54]	Phase II trial study N = 46 Age: 25–76 yrs; 45 Gy + boost 5.4 Gy in 1.8 Gy per fraction.	MTX	Median survival: 33 mo. 2-year OS 62 %	Dementia n = 6

Acbbreviations: HD-MTX – high-dose methotrexate; MTX - methotrexate; RT - radiotherapy; chemo - chemotherapy; SIB- simultaneous integrated boost; CR - complete response; OS - overall survival; PFS - progression-free survival.

## A practical approach to clinical practice

**Fig. 1:**
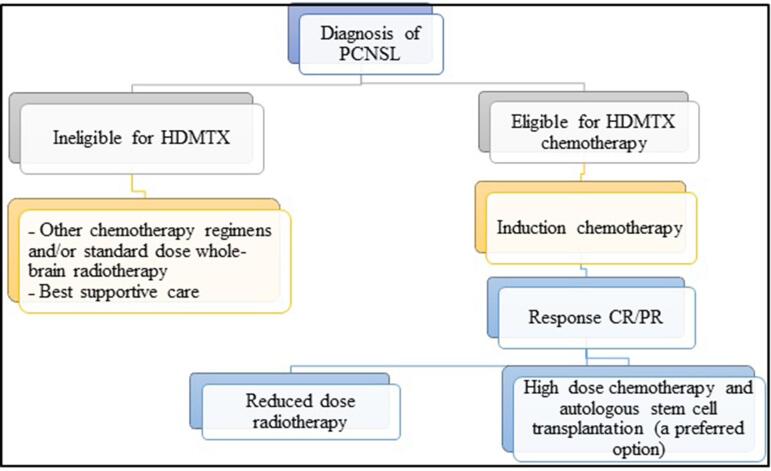
A schematic diagram represents the practical approach to the radiotherapeutic management of recently diagnosed patients. Abbreviations: PCNSL - primary central nervous system lymphoma; HDMTX - high dose methotrexate; CR - complete response; PR - partial response.

Based on emerging evidence and research, a practical and feasible algorithm for radiotherapy in the first-line treatment of patients with PCNSL was prepared and is displayed in Fig. 1 [5], [20], [21], [55]. HD-MTX is the drug of choice for PCNSL and is the backbone of induction chemotherapy regimens. While WBRT is an effective treatment, ASCT is a preferred option in the consolidation phase. In patients who are not eligible for high-dose MTX, alternative chemotherapy regimens and/or standard dose WBRT can be considered a treatment option. For elderly patients with poor performance status and significant comorbidities, best supportive care remains a reasonable alternative. Recommendations regarding the diagnostic pathway, imaging, biopsy, CSF analysis and choices of drug regimens were not part of this review.

## Target volumes and technique

Generally, PCNSL is a multifocal process thus a whole-cranial field is ideal and widely accepted. Because the optic nerve and the retina are connected to the cerebrospinal fluid circulation, cranial portal design always includes the posterior two thirds of the orbits to include both retinas even if there is no evidence of ocular disease, and the upper two cervical vertebrae. If the eyes are originally involved, the whole eyeballs should be included in the radiation field [10], [15], [17]. RT techniques have evolved dramatically over decades from a parallel opposed two fields technique, to 3D-conformal radiotherapy (3DCRT) techniques using multi-leaf collimators (MLC). For 3DCRT, the gross tumor volume (GTV) is the grossly visible tumor in post contrast T1 weighted MRI images. Positron emission tomography (PET) may be used for delineation to improve the accuracy if available. The whole brain and the sites harboring the potential microscopic disease including the posterior part of the orbit and the upper two cervical vertebrae, as mentioned above, are included in the clinical target volume (CTV). The planning target volume (PTV) is created as per standard protocols. The tumor boost is delineated adding a 1.5–2 cm margin to the gross tumor [10], [20]. The margin around the GTV to define the boost volume CTV seems to differ from one series to one another and the impact of these margins in terms of local control has not been assessed in prospective studies. If the boost is planned after chemotherapy, the post chemotherapy GTV can be used to contour the boost volume. 3DCRT is rather a preferred technique with the isocenter of the WBRT field being placed anteriorly near the orbit. In the modern era, there is a widespread use of intensity modulated radiotherapy (IMRT) which can be utilized for the simultaneous integrated boost technique if a higher dose is planned for the gross disease [20].

## Dose and fractionation

Severe forms of treatment-associated neurotoxicity have been reported with whole brain doses greater than 30 Gy, and patients treated with daily fractions <1.8 Gy are less likely to develop delayed toxicity [17], [26], [56]. Moreover, recent phase III trials have included reduced dose radiotherapy with MTX-based chemotherapy hence the total dose is recommended as follows.

• Standard dose WBRT as monotherapy − 36 Gy in 1.8 Gy per fraction with a sequential boost 9 Gy to the gross disease.
• Reduced dose WBRT as consolidation treatment − 23.4 Gy in 1.8 Gy per fraction in patients with complete response after induction chemotherapy; a sequential boost of 12.6 Gy in patients with partial response, or
• WBRT of 30 Gy in 1.5 Gy per fraction ± simultaneous integrated boost up to 40 Gy if the response was partial to systemic therapy.

## Future directions

One of the great challenges is to optimally treat patients who have residual disease after ASCT, and two trials examined the feasibility of RT in this setting. Specifically, in a multicenter phase II study of patients younger than 65 years of age, hyperfractionation was utilized following ASCT. Twenty-one patients proceeded with a total dose of 45 Gy (two doses of 1 Gy per day). 5-year overall survival probability was 87 %. However, late leukoencephalopathy was observed in some patients [57]. Further, a phase II trial investigated response adapted WBRT. Three out of 16 patients were eligible with a partial response after stem cell therapy, receiving 45 Gy. Although this yielded a better CR and a progression-free survival, neurotoxicity including leukoencephalopathy was a significant concern [58]. As WBRT after stem cell transplant has been associated with severe toxicities, future studies may investigate the feasibility of small volume stereotactic radiotherapy to the residual disease after high dose chemotherapy or as a bridge treatment between induction chemotherapy and ASCT [48].

Phase III NRG Oncology CC001 study hypothesized that hippocampal avoidance (HA) using IMRT could preserve cognition when WBRT was employed in brain metastasis. The risk of cognitive failure was significantly lower in patients who received HA-WBRT plus memantine, and they reported less difficulty with remembering things and speaking at 6 months. This innovative approach better preserves cognitive function and patient-reported symptoms [59]. The multifocal nature of PCNSL may not allow the adoption of HA-WBRT in the treatment but memantine can easily be incorporated with WBRT and future trials may examine this.

## Conclusion

In patients aged 70 years or younger, induction chemotherapy followed by consolidative high-dose chemotherapy plus ASCT or WBRT has been associated with a significant improvement in overall survival. ASCT is the most preferred treatment option as WBRT, especially with the dose of greater than 30 Gy, is occasionally associated with risk of permanent, irreversible neurotoxicity. However, the emerging evidence indicates low rates of unwanted late side effects and favorable treatment outcomes for low-dose radiotherapy in combination with chemotherapy. It seems clear that reducing the whole brain dose and boosting higher doses to the gross tumor may be considered a treatment option and be integrated into clinical practice if radiotherapy is chosen as a consolidative regimen. The prognosis for primary CNS lymphoma remains generally poor and a significant proportion of patients fails to attain prolonged disease control despite dramatic advancements in chemotherapy and radiotherapy. More research and phase III randomized trials are warranted to improve the effectiveness and efficiency of therapy, which includes advanced radiotherapy techniques and a combination of targeted therapy, immunotherapy, and low-dose radiation.

## Declaration of Competing Interest

The authors declare that they have no known competing financial interests or personal relationships that could have appeared to influence the work reported in this paper.
